# Cerebrospinal fluid metabolomic and postpartum depression: A Mendelian randomization study

**DOI:** 10.1097/MD.0000000000045489

**Published:** 2025-10-24

**Authors:** Minping Hong, Qin Xu, Xiaowen Huang, Junyan Wang, Zhenyi Ma

**Affiliations:** a Jiaxing Hospital of Traditional Chinese Medicine, Nanhu District, Jiaxing, Zhejiang Province, China.

**Keywords:** cerebrospinal fluid, Mendelian randomization, metabolomics, postpartum depression

## Abstract

Postpartum depression (PPD) is a multifaceted mental health disorder manifesting as enduring sadness, anxiety, and exhaustion following childbirth. Emerging evidence points to a metabolic dimension in its pathology. Our research examines the causal links between cerebrospinal fluid (CSF) metabolites and PPD via Mendelian randomization (MR). A bi-directional MR framework was applied to explore the causative connections between 338 CSF metabolites and PPD. The study harnessed data from 2 targeted genome-wide association studies: one encompassing single nucleotide polymorphism data from mothers diagnosed with PPD, and another concerning CSF metabolite profiles, both centered on European descendants. Instrumental variables from these datasets were meticulously selected to enhance MR analysis’ robustness. Our integrated approach provided a profound exploration of the genetic underpinnings influencing CSF metabolites related to PPD. Statistical analyses employed methods like inverse variance weighting, the weighted median, and mode-based estimation to fortify the causal inferences drawn from the associations. PPD was characterized according to diagnostic standards sanctioned by the FinnGen study’s clinical expert panels, incorporating insights from leading domain specialists. Our MR investigation pinpointed several CSF metabolites potentially linked to PPD. Notably, metabolites such as 3-hydroxy-3-methylglutarate, 3-methoxytyrosine, and argininosuccinate appeared protective, whereas arachidonate, benzoate, and carnitine correlated with heightened risk. The findings demonstrated consistency across diverse MR methodologies, affirming a significant linkage. This investigation underscores the potential of CSF metabolomics in decoding PPD’s etiology. Identifying particular metabolites associated with the disorder enhances our understanding of its underlying mechanisms and fosters avenues for future research into tailored therapeutic strategies.

## 1. Introduction

Postpartum depression (PPD) represents a significant mental health challenge that predominantly impacts mothers following childbirth.^[1,2]^ The latest review shows that the global prevalence of PPD symptoms is about 17%, which is higher in low- and middle-income countries, suggesting a high public health burden.^[3,4]^ It is characterized by a persistent state of sadness, anxiety, and tiredness that can severely affect the mother’s ability to care for herself and her newborn.^[5,6]^ This condition not only jeopardizes the health of the mother but also influences the emotional and developmental wellbeing of the child.^[7]^ Although the exact causes of PPD are still unclear, emerging studies are now investigating the involvement of metabolic pathways in its onset. In particular, recent research has suggested that variations in cerebrospinal fluid (CSF) metabolites may play a role in the development and severity of PPD, indicating a potential metabolic aspect to the condition.^[4,8,9]^ However, the direct causal relationships in these findings remain to be conclusively determined.

CSF metabolomics, which involves detailed analysis of metabolites within this biological fluid, has become an insightful tool for dissecting the complex interactions between genetic predispositions, environmental exposures, and disease mechanisms.^[10–12]^ In terms of biological mechanisms, previous studies suggest that PPD is associated with changes in the chemical environment of CSF. Perinatal CSF metabolomics studies reported that medium- and long-chain fatty acids were significantly associated with PPD symptoms with metabolites of tryptophan metabolic pathway, including capric acid, dodecanoic acid, arachidic acid, behenic acid, and L-tryptophan, which had good discriminative performance and had potential for identifying high-risk populations.^[4]^ Meanwhile, a systematic review summarized the clues of PPD-related CSF metabolism abnormalities, highlighting the importance of fatty acids and key pathways such as tryptophan/kynurenine.^[13]^ In addition, oxytocin at CSF levels is associated with PPD manifestations, suggesting the involvement of neuropeptide signaling. In women with persistent pain and PPD after cesarean section, plasma/CSF inflammatory factors were also altered, suggesting that neuroinflammation and metabolic networks may be intertwined to affect emotion regulation.^[8]^ However, most of the above studies are cross-sectional, small-sample, or candidate marker designs, making it difficult to clarify causality and systematically cover all CSF metabolite profiles.

Adopting Mendelian randomization (MR) offers a distinct advantage in this research context. MR leverages genetic variants as instrumental variables (IVs), offering a robust way to explore causal relationships between exposures and health outcomes while bypassing the usual biases inherent in observational studies. Our research applies the MR approach to examine whether there is a causative link between CSF metabolite alterations and PPD. This initiative positions our study at the forefront of this emerging research domain, aiming to deepen our understanding of PPD and expand potential therapeutic interventions.

## 2. Materials and methods

### 2.1. Study methodology

Utilizing a bi-directional MR approach, our investigation explored the putative causal links between 338 CSF metabolites^[14]^ and PPD.^[14]^ This MR methodology leverages genetic variations as IVs to infer causality, adhering to 3 fundamental principles:

1. Direct association: there exists a direct and specific association between genetic variations and the exposure under study.
2. No pleiotropy or confounding: the genetic variants used do not share a relationship with any potential confounders that could affect the interaction between the exposure and the outcome.
3. Instrument exclusivity: the influence of the genetic variations on the outcome occurs solely through their effect on the exposure, with no other pathways involved.

### 2.2. Data acquisition for PPD studies from genome-wide association studies (GWAS)

The statistical data for PPD were obtained from the GWAS catalog (https://gwas.mrcieu.ac.uk/), drawing from a dataset comprising 67,205 mothers. This dataset includes 7604 diagnosed cases and 59,601 controls, all of whom are of European ancestry. The criteria for diagnosing PPD were based on the FinnGen study’s established clinical standards.^[15]^ The FinnGen study provides a robust framework for the consistent and accurate definition of medical conditions, contributing to the reliability of our diagnostic approach.^[15]^

### 2.3. Sources of GWAS data for 338 CSF metabolites

For the 338 CSF metabolites, data were extracted from a comprehensive study by Daniel et al, as listed in Table S1, Supplemental Digital Content, https://links.lww.com/MD/Q472, focusing similarly on a European demographic.^[14]^

### 2.4. IV selection and validation

IVs were chosen based on a *P*-value threshold of 1 × 10^−5,[16–18]^ facilitated by the “TwoSampleMR” R package,^[19]^ To manage linkage disequilibrium, we applied a clumping threshold (*r*^2^ < 0.001, distance > 10,000 kb). Instrument strength was assessed using *R*^2^ and *F* statistics, excluding any single nucleotide polymorphisms (SNPs) with an *F*-statistic under 10 to prevent bias from weak instruments:


$$\begin{array}{l} R^{2}=\frac{2\beta_{exposure}^{2}eaf_{exposure}\left(1-eaf_{exposure}\right)}{2\beta_{exposure}^{2}eaf_{exposure}\left(1-eaf_{exposure}\right)+2{se}_{exposure}^{2}samplesize_{exposure}eaf_{exposure}\left(1-eaf_{exposure}\right)} \end{array}$$

$$\begin{array}{l} F=\frac{R^{2}\left(samplesize_{exposure}-2\right)}{1-R^{2}} \end{array}$$

In these formulae:

- β_exposure_ signifies the beta coefficient corresponding to the exposure variable;
- eaf_exposure_ denotes the effect allele frequency linked to the exposure;
- se_exposure_ outlines the standard error associated with the exposure;
- sample size_exposure_ specifies the total number of participants in the exposure cohort.

To enhance the reliability of our analysis, we only included SNPs with an *F*-statistic >10, thereby eliminating those with weaker instrumental value. This step helps prevent weak instrument bias, which is known to elevate type I error rates and compromise the accuracy of the results.^[20,21]^

### 2.5. Statistical approach

Utilizing R software (version 4.3.1; R Foundation for Statistical Computing, Vienna, Austria), our analysis incorporated multiple MR techniques, including inverse variance weighting (IVW),^[22]^ and mode-based estimation.^[23]^ We evaluated heterogeneity using the Cochran *Q* statistic and tested for potential pleiotropy through MR-Egger and MR-pleiotropy residual sum and outlier tests,^[24]^ supported by funnel plot analyses to confirm the robustness of our findings. In summary, the evaluation of CSF metabolites for their causal links to PPD was rigorously conducted, adhering to stringent criteria: achieving statistical significance in the primary analysis (IVW derived *P* < .05 and FDR < 0.8). In the initial screening phase, we applied a more lenient FDR threshold of < 0.8. This choice was made to increase sensitivity and reduce the likelihood of missing metabolites with small effect sizes that could still hold biological relevance. Although this relaxed threshold may increase the risk of false positives, it allows us to retain signals that may be biologically meaningful but have smaller effects; exhibiting consistent effect direction and magnitude across all applied MR methods; and demonstrating no significant heterogeneity or evidence of horizontal pleiotropy in the MR analyses.

## 3. Results

### 3.1. Exploration of CSF metabolites and PPD

Of 17,463 SNPs utilized as IVs (Table S2, Supplemental Digital Content, https://links.lww.com/MD/Q472), our MR analyses (Table S3, Supplemental Digital Content, https://links.lww.com/MD/Q472) pinpointed 7 key metabolites correlating with PPD. The analysis yielded:

Protective effects: 3-hydroxy-3-methylglutarate (OR: 0.81, *P* = .02), 3-methoxytyrosine (OR: 0.93, *P* = .01), and argininosuccinate (OR: 0.94, *P* = .01).

Potential risk factors: arachidonate (20: 4n6) (OR: 1.04, *P* = .03), benzoate (OR: 1.06, *P* = .03), and carnitine (OR: 1.24, *P* = .001).

**Figure 1.**
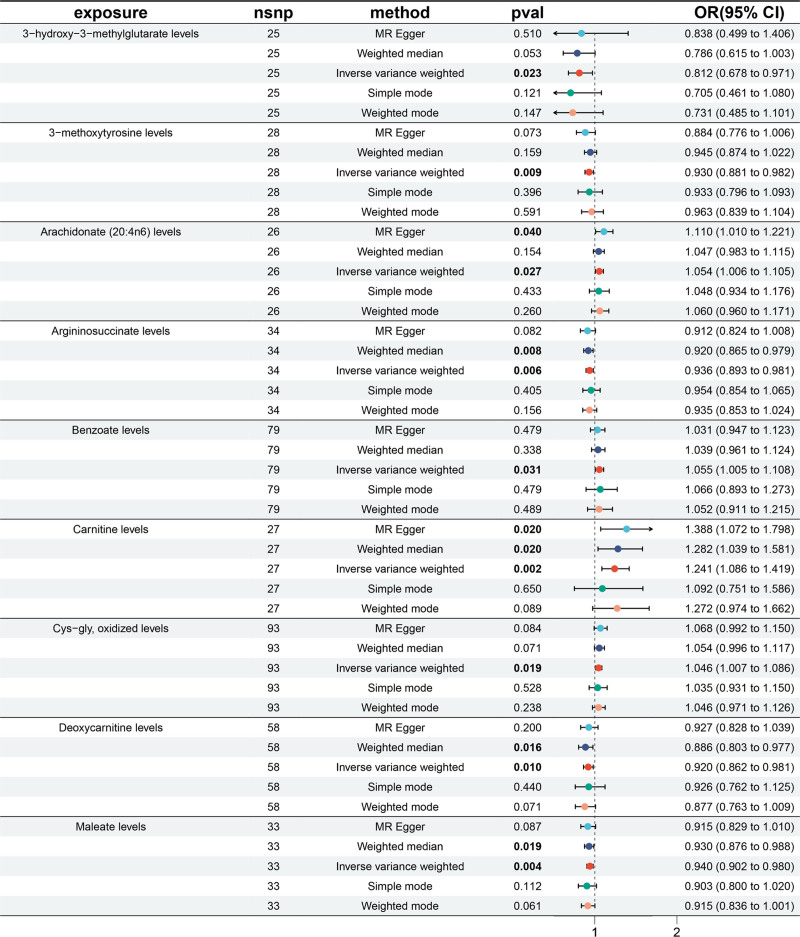
Forest diagrams displaying causal relationships between CSF metabolites and postpartum depression. The plots use inverse variance weighting (IVW) to illustrate the findings, with details on the confidence intervals (CIs) and the number of single nucleotide polymorphisms (nSNPs) included in each MR analysis. *P*-values < .05 are considered significant. CSF = cerebrospinal fluid.

**Figure 2.**
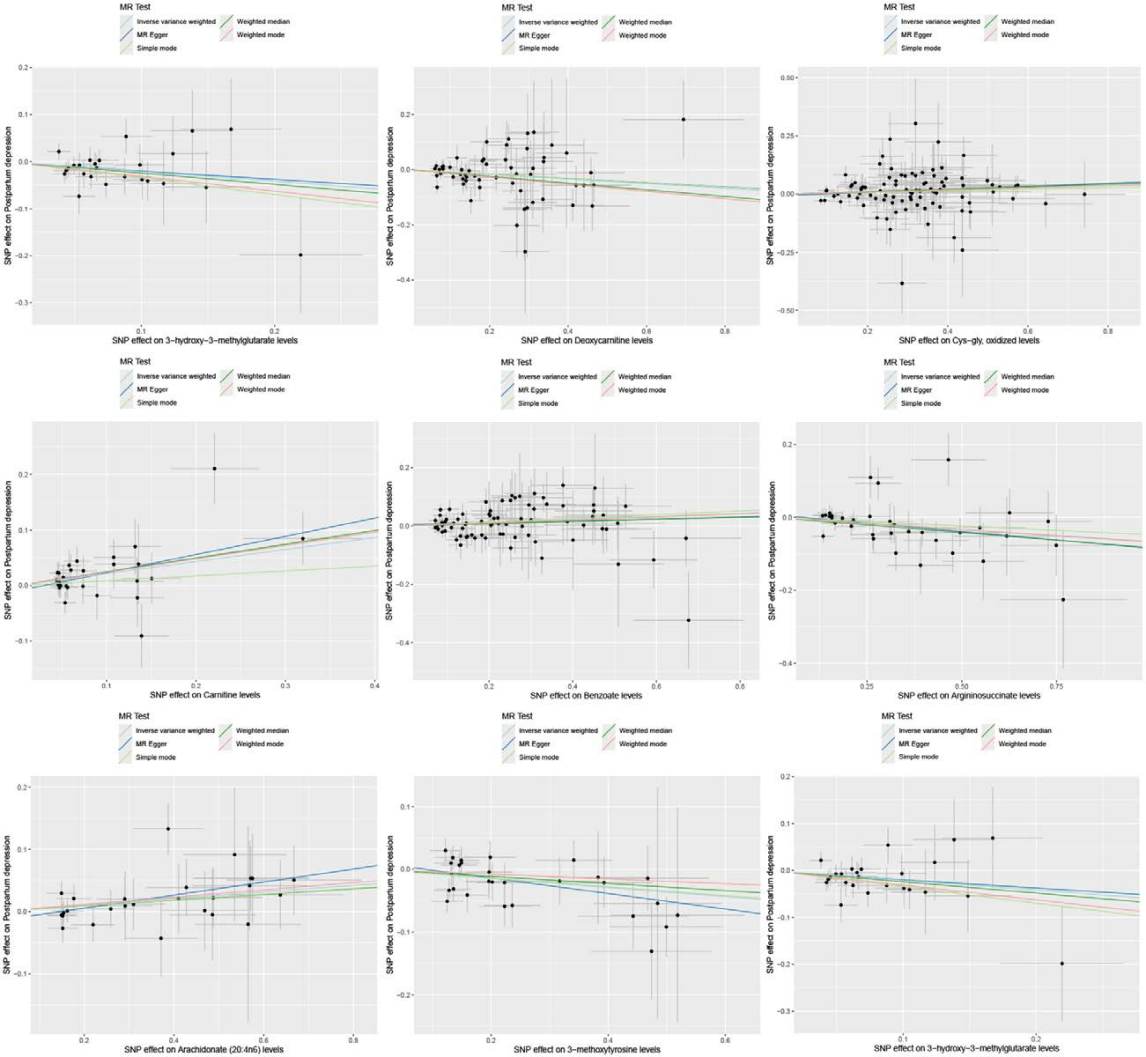
Scatter plots for the causal association between CSF metabolites and postpartum depression. CSF = cerebrospinal fluid.

**Figure 3.**
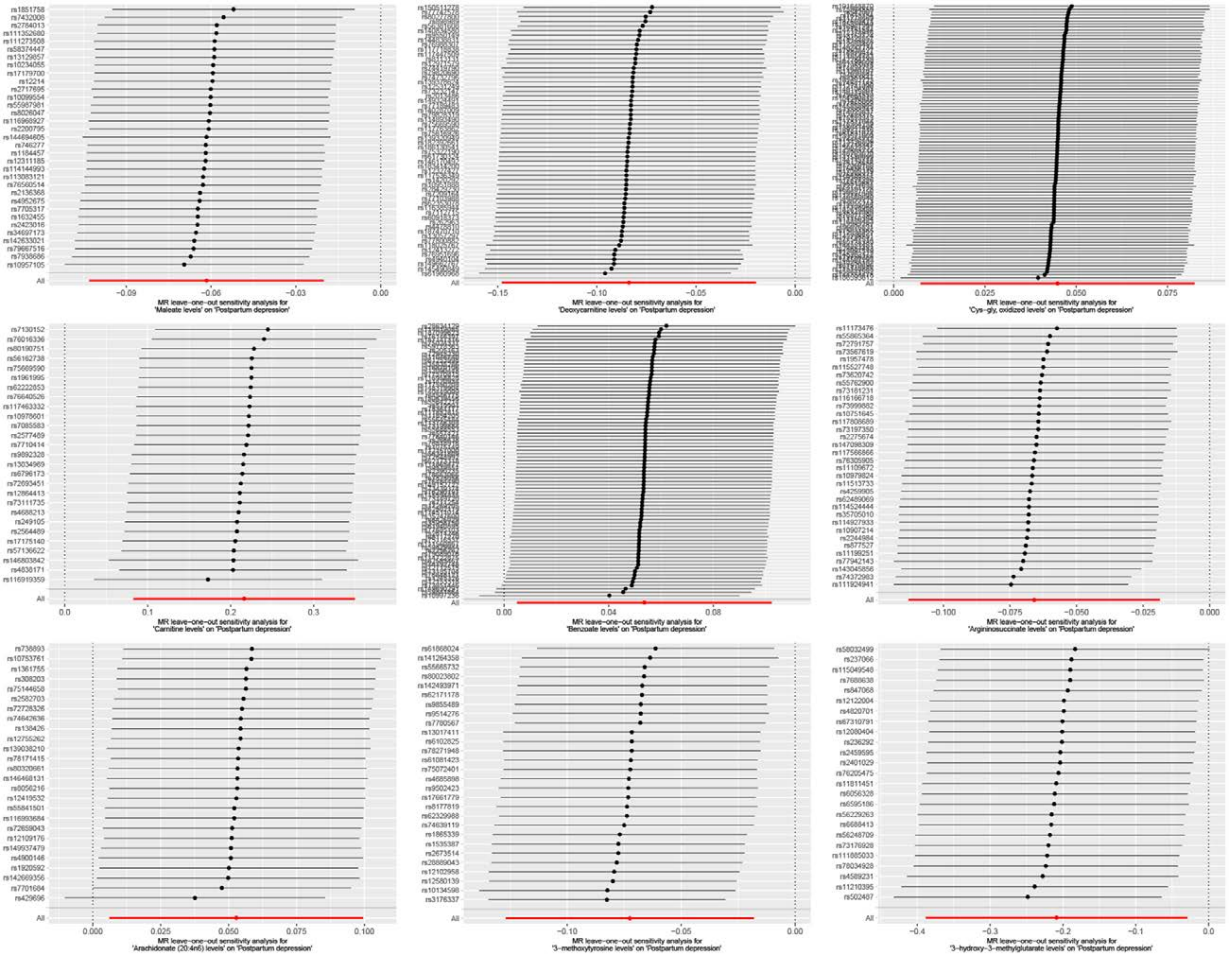
Leave-one-out sensitivity plots demonstrating the causal link between CSF metabolites and postpartum depression. These plots confirm the robustness of our results, showing consistent causal estimates even when individual genetic variants are removed from each analysis. This consistency underscores the reliability and stability of our findings. CSF = cerebrospinal fluid.

The forest plot (Fig. 1) summarizes point estimates and 95% confidence intervals from each MR method, highlighting directionally consistent effects across models. Scatter plots of SNP-exposure versus SNP-outcome effects (Fig. 2) illustrate the causal slopes for each metabolite, while leave-one-out analyses (Fig. 3) indicate that no single instrument drives the results materially.

The Cochran IVW *Q* test, detailed in Table S4, Supplemental Digital Content, https://links.lww.com/MD/Q472, showed no significant heterogeneity among the IVs used (*P* > .05). Additionally, the MR-Egger regression intercept, presented in Table S5, Supplemental Digital Content, https://links.lww.com/MD/Q472, did not indicate any significant directional horizontal pleiotropy (*P* > .05). The MR-pleiotropy residual sum and outlier global test, results outlined in Table S6, Supplemental Digital Content, https://links.lww.com/MD/Q472, also confirmed the absence of significant outliers, suggesting minimal horizontal pleiotropy affecting the relationship between CSF metabolites and PPD (*P* > .05). The robustness of these results, maintained even after the exclusion of individual genetic variants from the analyses, highlights the reliability and stability of our findings.

## 4. Discussion

In this study, we employed a bi-directional MR approach combined with GWAS data to explore the potential causal relationships between 338 CSF metabolites and PPD. Through a rigorous MR framework, we identified several CSF metabolites associated with PPD. Specifically, 3-hydroxy-3-methylglutarate, 3-methoxytyrosine, and argininosuccinate demonstrated protective effects against PPD, while arachidonate, benzoate, and carnitine were associated with an increased risk. These novel findings significantly enhance our understanding of the pathophysiology of PPD, emphasizing the importance of CSF metabolites in disease risk.

In our literature review, we found that some studies have reported associations between peripheral blood metabolites and PPD, but research on CSF metabolites is relatively scarce.^[25–27]^ In our study, some CSF metabolites, such as arachidonate and benzoate, have been mentioned in the context of other psychiatric disorders, but their direct association with PPD has been rarely reported.^[28,29]^ For instance, sodium benzoate, as an inhibitor of D-amino acid oxidase, has been studied in various psychiatric disorders, including schizophrenia and depression. It modulates NMDA receptor function by increasing synaptic levels of D-serine, a co-agonist of the NMDA receptor.^[30,31]^ This modulation is crucial for mood regulation and cognitive functions, highlighting the potential impact of sodium benzoate on psychiatric conditions.^[28]^

Similarly, other CSF metabolites identified in our study, such as 3-hydroxy-3-methylglutarate and 3-methoxytyrosine, although not directly associated with PPD in the current literature, may have indirect effects through their roles in other metabolic pathways. For example, 3-methoxytyrosine is a product of the dopamine metabolic pathway, which plays a crucial role in mood regulation.^[32]^ While the roles of these CSF metabolites are not yet fully understood, they merit further investigation for their potential impact on PPD.

A significant advantage of our study is the inclusion of extensive CSF metabolite GWAS data, an approach not commonly seen in prior research on PPD. The MR technique applied here robustly supports the investigation of causal links between CSF metabolites and PPD. By leveraging this methodology, we not only enhance our comprehension of the disorder but also pave the way for novel therapeutic interventions.

Despite its strengths, our research has certain constraints. Primarily, the scope of our sample size and its diversity are limited, which suggests that our results should be confirmed in more extensive and varied populations. Additionally, although MR helps mitigate issues of confounding and reverse causation, its effectiveness hinges on the strength of the genetic IVs used. Future research should therefore expand the number of participants and incorporate a broader array of genetic variants to improve the reliability and applicability of our findings.

Our research offers fresh perspectives on the metabolic underpinnings of PPD, especially through the study of CSF metabolites, potentially leading to innovative treatment approaches. It is crucial that future studies confirm these metabolites’ roles in the development of PPD and investigate their possible therapeutic benefits. Moreover, to gain a comprehensive understanding of the biological foundations of this multifaceted disorder, further research should encompass a wider variety of metabolites and extend to more diverse populations.

## 5. Conclusions

Our investigation, which combined extensive CSF metabolite GWAS data with MR techniques, revealed notable links between certain CSF metabolites and the likelihood of developing PPD. We identified specific metabolites associated with both elevated and reduced risks of the condition, providing novel insights into its metabolic foundations.

## Author contributions

**Data curation:** Xiaowen Huang.

**Formal analysis:** Xiaowen Huang.

**Writing – original draft:** Minping Hong, Qin Xu.

**Writing – review & editing:** Junyan Wang, Zhenyi Ma.

## Supplementary Material


